# Decoding choice and mutual information on a moment-by-moment basis from single neurons in rat prefrontal cortex

**DOI:** 10.1186/1471-2202-12-S1-P54

**Published:** 2011-07-18

**Authors:** Rick L Jenison, Craig W Berridge, David M Devilbiss

**Affiliations:** 1 Psychology Department, University of Wisconsin, Madison, WI 53705, USA

## 

Prefrontal cortex (PFC) neurons participate in representing a variety of stimuli, events, and processes that occur during the execution of behavioral tasks including spatial relationships as well as decision- and reward-related processes. We tested rats in a T-maze, delayed-alternation, decision-making task while simultaneously recording multiple single-unit spike trains from the prelimbic region of PFC. Animals were trained to navigate down the runway of the maze and choose one of two arms opposite to the one previously visited for food rewards. We used a recursive Bayesian approach to investigate the extent to which decision states could be decoded from the spike counts from neuronal ensembles in PFC at the time of decision-making. This approach estimates a full posterior probability distribution over states as a function of the spiking activity.

We estimated the Bayesian model using a boot-strap particle filter [1]. The particle filter is useful because it provides a computationally tractable approximation of the spike-train and task-related state probability distributions without assumptions of linearity or being Gaussian. Particle filtering starts with a collection of particles, which can be thought of as a cloud of guesses of the task-related state *x*_t_. Importance weights *W*_t_ are then assigned to each guess according to how likely a neural event *y*_t_ is, given the particular guess *x*_t_. By resampling we obtain a new set of particles that, in a certain sense, is consistent with the likelihood of generating *y*_t_ from *x*_t_. Additionally, these resampled particles can be regarded as a draw from the approximate posterior probability distribution *p*(*x*_t_ | *y*_t-1_) of task-related states. Calculating the expectation of the posterior probability distribution yields the point estimate of the state as the process iterates through time. To investigate the contribution of individual neurons to the decoding process, mutual information was calculated on a moment-by-moment basis between the state distribution and the neuron’s probability distribution of firing.

The animal’s choice to turn left or right in order to obtain a reward was decoded during each trial based on training spike-train data from all other trials (leave-one-out cross-validation). For each neuron in the decoded ensemble, the history of spiking activity and its interaction with choice contributed to the spike-train probability model.

The expected value calculated over 1000 binary particles of the filter diffused toward the observed behavioral choice in 85% and 76% of the trials of two animals. For trials where a behavioral error was observed (same arm choice on adjacent trials), the mutual information declined between the state distribution and spike train distribution indicating an increase in independence between task-related states and probability of neural firing. These results indicate that the particle filter accurately decoded the future behavioral choice of the animal during the pickup and delay intervals in two animals tested.
